# Managing Primary Infertility in a Woman With Uterine Fibroids Utilizing Myomectomy and In Vitro Maturation (IVM) of Oocytes

**DOI:** 10.7759/cureus.59257

**Published:** 2024-04-29

**Authors:** Radha Bondare, Jarul Shrivastava, Namrata Choudhary, Princee Tyagi, Shradha M Ulhe, Akash More

**Affiliations:** 1 Clinical Embryology, Datta Meghe Institute of Higher Education and Research, Wardha, IND

**Keywords:** myomectomy, icsi, ivm, uterine fibroid, infertility

## Abstract

This case report demonstrates the management of primary infertility in a couple: the male was 37 years old and the female was 32 years old. The female had a submucosal uterine fibroid. Later, the female underwent a myomectomy to remove submucosal fibroids in the uterus after two failed intrauterine insemination (IUI) cycles. After six months of her recovery period, she underwent ovum pickup for an in vitro fertilization (IVF) cycle. During the process of ovum pickup (OPU), four oocytes were retrieved: three in the metaphase one (M1) stage and one in the metaphase two (M2) stage. Subsequently, the couple underwent in vitro maturation (IVM) of oocytes, where the M1 stage oocytes were cultured for six hours. The M1 stage oocytes progressed to the M2 stage. These oocytes were then injected with sperm, which resulted in the formation of two blastocysts. These blastocysts were then cryopreserved for three months, and after three months, these frozen embryos were then transferred, leading to the successful conception. The case study evaluates a couple who suffered from infertility. This study includes a treatment of myomectomy and in vitro maturation.

## Introduction

Infertility is a condition occurring in males as well as in females and is defined by the failure to achieve pregnancy after 12 months or more of regular unprotected sexual intercourse [1]. Uterine fibroids (UFs) are one of the main causes of infertility. Uterine fibroids are abnormal benign tumors that develop at various sites of the uterus, varying in size and number. During the reproductive age of a woman, these smooth muscle benign tumors may exhibit different symptoms. Some common symptoms are menstrual bleeding, frequent urination, and discomfort during the menstrual cycle [1]. Additionally, women may experience various other symptoms, such as swelling or a feeling of fullness in the abdominal area, pelvic pressure, painful bowel movements, and bladder or bowel dysfunction leading to constipation. When menopause begins, over 70% of women are affected by common uterine fibroid tumors. About 25% of women with uterine fibroids experience severe symptoms that require medical treatment. The treatment options range from medication to surgical procedures, mainly depending on the severity of the symptoms and the individual's health [1]. During the diagnosis, fibroids can cause abnormally excessive bleeding (menorrhagia), pain, and the development of a mass and pressure [2]. Fibroids may also be associated with miscarriages or fertility problems. In addition, obesity, infertility, and long-term use of oral contraceptives are risk factors for the development of fibroids, which can also be asymptomatic. Infertility can occur when fibroids obstruct the fallopian tubes, contributing to infertility in 2-3% of women [2]. Abortions and infertility result from the presence of fibroids in certain cases, depending on their location within the uterus [2]. Uterine fibroids affect 70% of women during their lifetime and have a direct impact on their quality of life, health, and economic costs. However, there is still no fully effective conservative therapy for fibroids. Approximately 70% of uterine fibroids are treated through medical diagnosis, while the remaining 30% of patients undergo surgical treatment or interventional radiological procedures [3]. Gonadotrophin-releasing hormone (GnRH) is a hormone that modulates the pituitary gland in the brain. GnRH agonists and antagonists are used to stimulate the ovaries to produce more eggs. Both play a role in vitro fertilization (IVF) but achieve their purposes in distinct ways. Antagonist GnRH, like leuprolide acetate, works similarly to the body's natural GnRH. The pituitary gland is instructed to release luteinizing hormone (LH) and follicle-stimulating hormone (FSH). However, sometimes GnRH agonists prevent the pituitary gland from releasing these hormones. Due to the decline in estrogen levels, the ovaries delay the production of eggs until the ovary is stimulated again. GnRH antagonists, such as ganirelix acetate, promptly block pituitary production of FSH and LH . Consequently, GnRH antagonists also prevent premature egg release [4]. In vitro maturation (IVM) was first effectively implemented in 1991, resulting in a successful pregnancy. Since then, it has been successfully employed as an alternative to IVF in different procedures. IVM is an option in assisted reproductive technique [5]. Comparable to IVF, the IVM oocyte treatment can typically result in a clinical pregnancy rate of up to 35% in young women [6]. As the technique develops, IVM may eventually replace IVF in some circumstances as the pregnancy rate increases. It is a simpler alternative to standard IVF for several indications [6]. For those trying to preserve their fertility, especially in situations where regulated ovarian stimulation is not feasible, IVM of the human oocyte in matured oocytes is a flexible option. To enhance the chance of recovery in cancer patients, the IVM technique can be used in addition to ovarian tissue cryobanking [7].

## Case presentation

Patient information

A 32-year-old female, along with her 37-year-old husband, visited an infertility clinic for a consultation regarding their infertility. They had been married for three years and had been actively trying to conceive for the last two years. The female patient was a homemaker and the husband a farmer by profession. The couple had two unsuccessful intrauterine insemination (IUI) cycles in their past medical history. 

Medical/surgical history

The couple had a history of two unsuccessful IUI cycles at another center. There is no previous family history of any disease. The woman had a standard body mass index (BMI) of 22 kg/m^2^, and her husband had a normal BMI of 24 kg/m^2^. No specific medical or surgical history that may cause infertility has been observed, and the cause of infertility could be idiopathic.

Investigation and diagnosis

The male patient underwent semen analysis and the results showed a normal sperm count of 50 million/ml, which was above the reference range (15-20 million/ml). Normal motility was 75%, above the World Health Organization (WHO) reference range (30%). The female patient underwent ultrasonography to assess the functioning and structure of the uterus. The findings confirmed the presence of submucosal fibroids in the uterus. The female's anti-Müllerian hormone (AMH) level was 2.2 ng/ml, FSH level was < 7.3 IU/ml, and LH level was 5.4 IU/ml. This was the case of primary infertility. After transvaginal ultrasound, uterine fibroids were identified as a potential cause of infertility in the female, while semen analysis of the male partner was found to be normal.

Treatment

**Figure 1:**
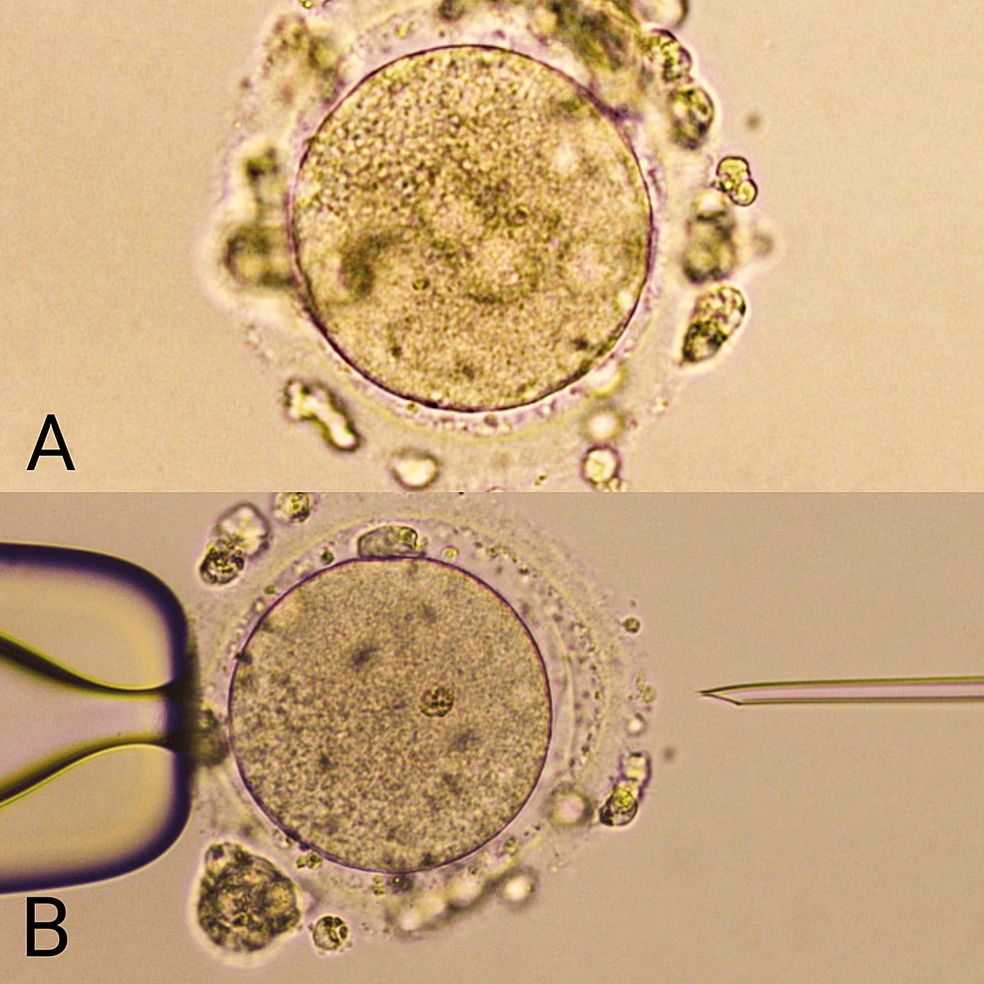
M1 oocytes converted into M2 oocytes after IVM treatment: (A) M1 oocytes retrieved during oocyte pickup (OPU); (B) M1 ooctyes converted into M2 oocytes after IVM treatment. IVM: in-vitro maturation

Firstly, the patient was advised to undergo a myomectomy to remove the fibroids, which was performed a week later. Following the surgery, the patient was prescribed leuprolide acetate 3.7mg and instructed to observe a six-month recovery period. After the recovery period, the patient underwent oocyte pickup 36 hours after administering the trigger human chorionic gonadotropin (HCG). Four oocytes were successfully retrieved from both ovaries: one oocyte in the metaphase II (M2 phase) and three in the metaphase I (M1 phase). The sperms were processed using the density gradient (DG) method. Subsequently, intracytoplasmic sperm injection (ICSI) was performed with metaphase II (M2 stage) oocytes and the male sperm. The remaining three M1 stage oocytes were cultured in IVM media for six hours. After six hours, two M1-stage oocytes reached the M2 stage as shown in Figure 1. Later, intracytoplasmic sperm injection (ICSI) was performed, and the injected oocytes were cultured under favorable conditions for the next 17-20 hours to evaluate fertilization. On the fifth day, two blastocysts were formed and frozen. After three months, frozen embryo transfers were carried out.

Follow-up

On the fifteenth day after embryo transfer (ET), the β-HCG test revealed positive results (1112mIU/mL). The patient was consulted regarding the significance of consistent care during pregnancy. Several medications containing progesterone and estrogen were prescribed to support the uterus. The health of the fetus was regularly monitored.

## Discussion

Uterine fibroids, also called leiomyomas, are benign smooth muscle tumors of the uterus that affect women of reproductive age. The pathogenesis, size, location, and symptoms of fibroids vary significantly. Some women experience menstrual disorders like hypermenorrhea and dysmenorrhea, while others may not have any symptoms at all. Their intensity and the symptoms may vary depending on the location and size of the fibroids. The most frequent initial symptom is heavy menstrual flow, which may result in anemia, exhaustion, or painful periods [1]. Fibroids can alter the receptivity of the endometrium, causing the cavity to swell. In as many as 27% of patients seeking reproductive assistance, there is a lack of research on the safety and efficacy of medical, surgical, and non-invasive techniques for treating fibroids in infertility treatment, as well as how fibroids affect pregnancy. Myomectomy is the most evidence-based treatment for fibroids in women who are trying to conceive, and it can increase patient pregnancy rates [8].

Laparoscopic myomectomy is considered to be a safe treatment with a very low failure rate and positive pregnancy outcomes when carried out by an experienced surgeon. In 1979, the first report of laparoscopic myomectomy appeared; it was confined to subserous fibroids [9].

Our case report demonstrates IVM as a proven and effective method for maturing oocytes. M1 stage oocytes (immature oocytes) were converted into M2 phase oocytes after the ICSI was performed. The fertilized egg cleaved, was implanted, and produced a successful pregnancy outcome, in addition to the successful fertilization that took place after the removal of the fibroids and M1 stage oocytes. Through the process of IVM, significantly improved results have been achieved. IVM is a useful technique for infertile couples and helps to preserve fertility within the moderate activation level of IVF, which, coupled with IVM of immature oocytes, is a possible complement to traditional type of stimulation IVF cycle treatment. It becomes the most optimal first-line treatment alternative [10].

## Conclusions

This case report shows successful treatment using IVM in a woman of reproductive age with primary infertility. The probable cause of her infertility was submucosal fibroids, which were removed via myomectomy. This case report demonstrates that using IVM as an oocyte maturation technique is beneficial. Later, the ICSI was carried out with good-quality sperms and oocytes, giving positive pregnancy outcomes.
